# Lung abscess without sepsis in a patient with diabetes with refractory episodes of spontaneous hypoglycemia: a case report and review of the literature

**DOI:** 10.1186/1752-1947-8-51

**Published:** 2014-02-13

**Authors:** Gotaro Toda, Midori Fujishiro, Tomohide Yamada, Nobuhiro Shojima, Hideyuki Sakoda, Ryo Suzuki, Toshimasa Yamauchi, Kohjiro Ueki, Takashi Kadowaki

**Affiliations:** 1 Department of Diabetes and Metabolic Diseases, Graduate School of Medicine, The University of Tokyo, 7-3-1, Hongo, Bunkyo-ku, Tokyo, Japan

**Keywords:** Hypoglycemia, Type 2 diabetes, Infection, Insulin resistance, Glucocorticoids

## Abstract

**Introduction:**

Hypoglycemia is a cause of considerable morbidity. Although hypoglycemia has been documented in the setting of septic shock and has been associated with higher mortality, hypoglycemia in infection without sepsis has not been reported in the literature.

**Case presentation:**

A 72-year-old Japanese woman treated with high-dose glucocorticoids for autoimmune hemolytic anemia, as well as intensive insulin therapy for type 2 diabetes, presented with severe hypoglycemia. A lung abscess was diagnosed by imaging studies and treated with intravenous antibiotics. Hypoglycemia spontaneously recurred during lung abscess exacerbations, despite appropriate de-escalation of antidiabetic therapy. Only mild sporadic episodes of hypoglycemia occurred after the lung abscess was controlled. Infection accompanied with malnutrition and immunosuppression, although in the absence of sepsis, may have contributed to hypoglycemia.

**Conclusions:**

Caution is warranted in the management of hypoglycemia in patients with diabetes with the conditions described here, that is malnutrition and immunosuppression, as infection may be a contributing factor.

## Introduction

Hypoglycemia is a cause of considerable morbidity, and is of major concern throughout the treatment of diabetes mellitus. Although infection is known to exacerbate insulin resistance and cause hyperglycemia, septic shock produces hypoglycemia. However, to the best of our knowledge, hypoglycemia in infection without sepsis has not been reported in the literature. Here, we present the case of a patient experiencing refractory episodes of spontaneous hypoglycemia in which lung abscess, although without sepsis, may have played a central role.

## Case presentation

A 72-year-old Japanese woman presented to a neighboring hospital with loss of consciousness due to severe hypoglycemia (plasma glucose 10mg/dl). She was transferred to our facility the following day. She had had type 2 diabetes, treated with insulin (basal-supported oral therapy) for three years before admission. Three months before admission, she was diagnosed with autoimmune hemolytic anemia (AIHA) and was started on 70mg oral prednisolone per day, which exacerbated her diabetes, necessitating intensive insulin therapy. By two weeks before admission, oral prednisolone was tapered to 50mg as her hemoglobin concentration had stabilized around 10g/dl. She experienced no episodes of overt hypoglycemia prior to hospitalization.

**Figure 1:**
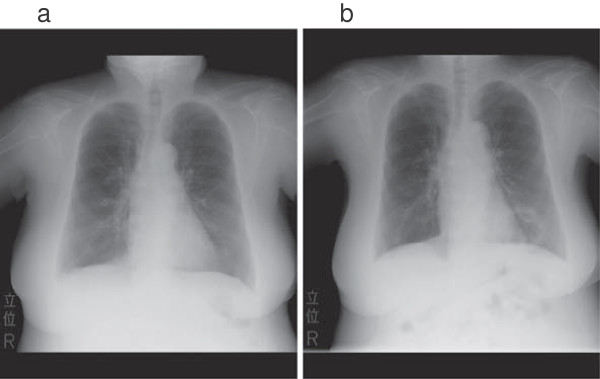
**Chest radiographs obtained one month before admission (a), and on admission (b).** A large hollow lesion in the lower left lung field was diagnosed as a lung abscess.

Our patient was obese with a body mass index of 31.2kg/m^2^. Her medications included insulin (27 units/day), and oral prednisolone (50mg/day). Her insulin dosage was 7 units of insulin aspart before breakfast, 4 units before other meals, and 12 units of insulin glargine at bedtime. Chest radiography on admission revealed a high-density area with a hollow lesion not detected the previous month (Figure 1), which was confirmed by computed tomography. A lung abscess was diagnosed and intravenous 4.5g piperacillin/tazobactam every 8 hours was started. Her sputum and blood culture test results were negative. Laboratory examinations showed elevated serum C-reactive protein (7.32mg/dl), and normal fasting adrenocorticotropic hormone (ACTH) (19.1pg/ml) and cortisol (10.6μg/ml) levels despite administration of 50mg of prednisolone per day. Her fasting and postprandial plasma C-peptide levels were low (fasting and postprandial plasma glucose and C-peptide levels were 221mg/dl, 0.5ng/ml and 293mg/dl, 0.4ng/ml respectively). Our patient’s kidney function was normal with a serum creatinine level of 0.76mg/dl. The hemoglobin concentration remained stable (10.5g/dl), and hemoglobin (Hb) A1c was stable at 7.6% (59.6mmol/mol). Her serum albumin was low (1.8g/dl). Her anti-insulin antibody test was negative (Table 1).

**Table 1:** Results of laboratory examinations on admission

White blood cells	18,700	/ul
Band cells	4.0	%
Segmented cells	93.0	%
Eosinophils	0.0	%
Hemoglobin	10.5	g/dl
Platelet count	294,000	/ul
Serum albumin	1.8	g/dl
Serum lactate dehydrogenase	432	U/L
Serum aspartate aminotransferase	18	U/L
Serum alanine aminotransferase	19	U/L
Serum blood urea nitrogen	21.9	mg/dl
Serum creatinine	0.76	mg/dl
Serum sodium	136	mEq/L
Serum potassium	4.7	mEq/L
Serum creatine kinase	31	U/L
Serum glycoalbumin	23.3	%
Serum C-reactive protein	7.32	mg/dl
Fasting adrenocorticotropic hormone	19.1	pg/ml
Fasting cortisol	10.6	ug/ml
HbA1c	7.6	%
Fasting plasma glucose	221	mg/dl
Fasting C-peptide reactivity	0.5	ng/ml
Postprandial plasma glucose	293	mg/dl
Postprandial C-peptide reactivity	0.4	ng/ml
Anti-insulin antibody	Negative	

HbA1c, hemoglobin A1c.

**Figure 2:**
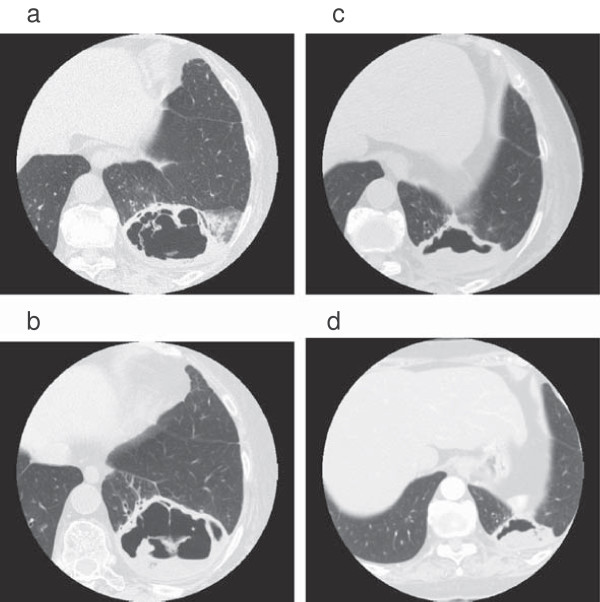
**Computed tomographic images of the lung abscess on the second (a), 31**^**st **^**(b), 79**^**th **^**(c), and 98**^**th **^**(d) days after admission.** The maximum diameters of the lesion were 60, 87, 60, and 50mm, respectively. As shown here, the lesion was largest around the 30^th^ day after admission.

**Figure 3:**
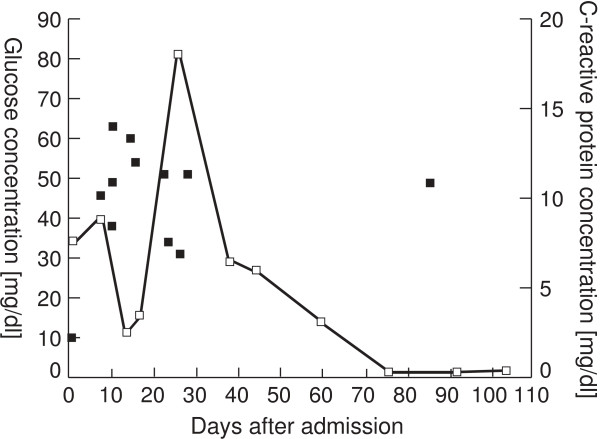
**Hypoglycemic episodes after admission.** Black squares represent glucose concentrations below 70mg/dl. Glucose concentrations were measured four or six times daily. White squares represent plasma C-reactive protein concentrations. Hypoglycemic episodes were mainly observed from admission to the 30^th^ day of hospitalization, during which the lung abscess was enlarging as shown in Figure 2.

Recurrent episodes of hypoglycemia continued after admission, and did not resolve until the 30th day of hospitalization, the point at which the lung lesion was largest (Figure 2, 8.7×7.0cm axially). Hypoglycemia was observed 16 times in the same period, with a maximum of three times in the same day (Figure 3). Episodes were observed before and after meals, and were not concentrated in a particular time of the day. After admission, her insulin dosage was reduced to 3 units of insulin aspart before meals, which was skipped when oral intake was insufficient. Her insulin glargine was not reduced, due to high levels of fasting plasma glucose. Oral therapy consisted of voglibose (0.2mg before meals), and buformine hydrochloride (100mg before breakfast). Buformine was discontinued shortly after admission. A dosage of 50mg oral prednisolone was constantly administered from two weeks before admission until episodes of hypoglycemia ceased to appear. Episodes of hypoglycemia were not observed as the lesion decreased in size after this point (Figure 2, 5.0cm axially on the 98^th^ day of hospitalization), and our patient was discharged from our hospital after two months of antibiotic treatment.

## Discussion

Possible causative factors of recurrent hypoglycemia in the present case included insulin use, malnutrition, and severe infection resulting from the lung abscess. Insulin was unlikely to have played a central role, as no episodes of hypoglycemia were observed under administration of the same dosages of insulin before admission. Insulin dosages were appropriately skipped or tapered according to the amount of oral intake in unsuccessful attempts to avoid hypoglycemia during periods where hypoglycemia was observed. Oral intake was approximately 1000kcal/day, which was inadequate (21kcal per ideal body weight). However, we speculate malnutrition was not central to the pathogenesis of hypoglycemia, as oral intake was similar before and after the episodes of hypoglycemia. The infection was likely to be severe and prolonged, as suggested by low serum albumin levels and a rise in C-reactive protein to a maximum of 18.03mg/dl on day 24, though symptoms of infection in this patient may have been masked by glucocorticoid use. The normal levels of fasting ACTH and cortisol raised the possibility of very strong secretory stimuli, such as severe infection, and/or hypoglycemia causing a limited ACTH response even under high prednisolone doses. Moreover, the absence of hallmarks of adrenal insufficiency (stable systolic blood pressure at around 120mmHg, normal levels of potassium and eosinophils throughout the patient’s course) led us to believe adrenal insufficiency was not central in the pathogenesis of hypoglycemia. Supported by the observation that hypoglycemia and the lung abscess had exhibited parallel courses, severe infection may have played a central role in the pathogenesis of recurrent hypoglycemia in this patient.

Previous studies showed immunocompromised hosts with lung abscess to be more likely to develop lung abscesses with positive cultures for aerobes, as compared with immunocompetent hosts (63% vs. 20%; P = 0.057) [1], and that patients with lung abscess who have diabetes are at increased risk for lung abscess with positive cultures for *Klebsiella pneumoniae* compared to patients without diabetes (odds ratio 4.3, 95% confidence interval, 1.0 to 18.4, P = 0.039) [2]. On these grounds, we speculate Gram-negative bacteria, such as *K. pneumoniae*, are candidates for the virulent organism in this patient receiving high-dose glucocorticoids in addition to having diabetes.

Hypoglycemia has been reported in 16.3% of patients with sepsis, and has been associated with higher mortality [3]. Extensive research has been conducted to elucidate the mechanisms of hypoglycemia in infection, using animal models injected with lipopolysaccharide (LPS). LPS induces hyperglycemia approximately 1 hour after injection into mice [4] followed by hypoglycemia within 6 hours [5]. Glycogen depletion, decreased gluconeogenesis, and increased glucose consumption have been suggested as mechanisms of hypoglycemia induction by LPS [5,6]. LPS inhibits insulin signaling *in vivo*, which leads to decreased glycogen synthesis, increased glycogenolysis and also increased gluconeogenesis [7]. Euglycemic hyperinsulinemic clamp studies on animal models of LPS-induced hyperglycemia support this view, revealing an increase in hepatic glucose output, possibly contributing to glycogen depletion [4]. LPS-induced cytokines such as interleukin (IL)-1β and tumor necrosis factor (TNF) α impair the glucagon-induced increase in gluconeogenesis in animal models [8]. LPS also downregulates cytoplasmic glucocorticoid receptors in the livers of mice, resulting in inhibition of the glucocorticoid-induced increase in hepatic gluconeogenesis [5]. Negative blood cultures on multiple occasions in the present case were not suggestive of sepsis. However, taking the above observations together, we speculate that hypoglycemia can be induced by severe prolonged infection without signs of sepsis in settings of malnutrition and immunosuppression, even with high-dose glucocorticoid treatment, as in the present case.

## Conclusions

Clinicians should exercise particular caution managing hypoglycemia in settings of malnutrition, immunosuppression, and glucocorticoid use, as severe bacterial infection may be involved in its pathogenesis.

## Consent

Written informed consent was obtained from the patient for publication of this case report and any accompanying images. A copy of the written consent is available for review by the Editor-in-Chief of this journal.

## Competing interests

The authors declare that they have no competing interests.

## Authors’ contributions

GT wrote the manuscript. GT, MF, TY, NS, HS, RS, TY, KU, and TK interpreted the patient data and contributed to the discussion. TK was the guarantor. All authors read and approved the final manuscript.
